# Awareness of Diabetic Retinopathy Among Patients With Type 2 Diabetes Attending the Outpatient Clinic in the General Hospital in Rabigh, Saudi Arabia: A Cross-Sectional Study

**DOI:** 10.7759/cureus.28235

**Published:** 2022-08-21

**Authors:** Iman Wahby, Fatma Albeladi, Abdulrahman Abukhudair, Shahad Alyoubi, Safa Alyoubi, Alanoud Alotaibi, Atheer Albeladi

**Affiliations:** 1 Community Medicine, King Abdulaziz University, Jeddah, SAU; 2 Nephrology, Faculty of Medicine, King Abdulaziz University, Jeddah, SAU; 3 Medicine and Surgery, Faculty of Medicine, King Abdulaziz University, Rabigh, SAU

**Keywords:** diabetic eye disease, retinopathy, saudi arabia, diabetes mellitus., diabetic retinopathy, awareness

## Abstract

Introduction: Diabetic retinopathy (DR) is a common microvascular complication of type 2 diabetes (T2D) and a major cause of blindness. DR awareness is important for early identification and management in patients with T2D. This study aimed to estimate the level of awareness of DR and its risk factors among patients with T2D in Saudi Arabia.

Methods: We conducted a cross-sectional study to analyze data collected from 291 patients with T2D attending outpatient clinics in the General Hospital in Rabigh during 2020-2021. We collected demographic information and level of awareness about T2D and DR.

Results: Among 291 patients with T2D, 42.3% had T2D for more than five years, and 37.8% had T2D for two to five years. In our study population, 32.3% of participants obtained high school education, and 42.3% had moderate income. Over half of respondents (56.4%) had their last eye exam within the past year, and 68.4% of participants believed high blood glucose levels might cause vision problems. The mean ± standard deviation of the DR awareness score was 7.23 ± 2.74. Most participants had moderate level of awareness (39.5% of participants), 31.6% had good level of awareness, and 28.9% had poor level of awareness about T2D and DR. Participants without DR or who had DR for less than two years and those who had their eyes checked by a doctor last year had a significantly higher DR awareness level.

Conclusion: We asked patients with T2D to assess their level of DR awareness. Most patients had moderate awareness levels, indicating a need for improved awareness of T2D complication on retina and treatment options. Patients should also be motivated for retinal screening to reduce the risk of visual complications. Furthermore, DR screening programs should not be limited to eye care centers. Improved awareness and access to screening programs will help patients and their healthcare providers achieve optimal outcomes in prevention of DR.

## Introduction

Diabetes mellitus is characterized by chronic hyperglycemia and impaired carbohydrate metabolism due to a complete or partial lack of insulin secretion and/or action [1]. Type 2 diabetes (T2D) is the most common type of diabetes, accounting for 90%-95% of all patients with diabetes [2]. Global estimates indicate that 439 million people will be diagnosed with T2D by 2030 [3]. Most cases of T2D are caused by the interplay of genetic, environmental, and behavioral risk factors [4].

Diabetes is becoming more common in high- and low-income countries [5]. Saudi Arabia was rated seventh out of 10 highest countries in prevalence of diabetes in 2013, with 24% of adults aged 20-79 years diagnosed with diabetes, and the incidence is expected to climb [6]. Diabetes can damage organ systems resulting in serious problems over time, such as retinopathy, neuropathy, and nephropathy [1]. Because of its ocular consequences, T2D is a leading cause of blindness worldwide [7].

Diabetic retinopathy (DR) is one of the diabetic complications causing blindness around the world [8]. DR consists of early nonproliferative DR (NPDR) and advanced proliferative DR (PDR). The clinical features classify NPDR, such as retinal hemorrhages, microaneurysms, venous caliber changes, and intraretinal microvascular abnormalities. PDR is differentiated by pathologic preretinal neovascularization [9]. Studies in Saudi Arabia reported that the prevalence of DR is 16.7%-31% [10-11].

The duration of diabetes, poor blood glucose management, and the presence of other systemic disorders such as hypertension (HTN) are risk factors for DR [12-13]. Early screening, detection, and treatment of DR among patients with T2D require a strong awareness of DR and its risk factors to avoid potential visual damage [13-14]. Lack of knowledge regarding T2D, DR, the need for routine eye exams, and treatment advantages can result in poor adherence to recommendations, referral delays, and presentation with advanced DR [15-16]. This study aimed to assess the level of DR awareness and its associated risk factors among patients with T2D in Rabigh, Saudi Arabia.

## Materials and methods

We conducted a cross-sectional study in the general hospital in Rabigh having patients with T2D diagnosed at least one year before the study started. The study included any Saudi adults age 25 or older with any degree of education, male or female. The study excluded patients with type 1 diabetes, patients younger than age 25, or pregnant patients. This study was approved by the institutional review board of King Abdulaziz University (Reference No 568-19). We met the patients in person in the clinic and used a self-constructed questionnaire from previous study which validated by revision of two community medicine and one internal medicine consultant to collect the data. The questionnaire was divided into two sections; the first concerned patient demographic information (e.g., age, gender, nationality, place of residence, and education level), and the second measured personal level of awareness of T2D and DR.

Twelve questions assessed participant level of awareness regarding DR using Likert-Type Scales [17]. Each right or positive answer was given a score of “1,” and a score of “0” was given for the wrong and “I don’t care” answers. The highest possible score is 12. Participants were classified according to their level of awareness into those having a poor level of awareness (i.e., scores < 6), a fair level of awareness (scores of 6-9), and a good level of awareness (scores of 10-12).

The data were coded, entered, cleaned, and analyzed using IBM SPSS Statistics for Windows, Version 22.0. (IBM Corp., Armonk, NY). Qualitative variables were presented using frequencies and percentages, while quantitative variables were shown as the mean ± standard deviation. A Chi-squared test was used to compare between two qualitative variables. We used a 95% confidence interval, and p<0.05 was considered statistically significant. And there was no multivariate analysis test.

## Results

Table 1 presents the distribution of 291 participants according to demographic data. The most common age range was 46-55 years (31.6%), 56.4% of the study population was male, 91.1% had a Saudi nationality, and 74.2% were from the Rabigh region. Approximately one-third (32.3%) of the participants had a high school education, 74.9% were married, 42.3% had a monthly income of 5000-10000 Saudi Riyals (SR), and 56.4% were employed. Also, 10% of participants were current smokers, and 10% engaged in regular physical activity. Most of them (60.8%) had chronic diseases, the most common of which was HTN; 44.7%. Approximately 43% of the participants were overweight [mean body mass index (BMI): 26.88 ± 4.57 kg/m2].

**Table 1:** Study population demographic data, type 2 diabetes, diabetic retinopathy, and timing of eye examinations (N=291). SR, Saudi Riyals; HTN, hypertension; CVD, cardiovascular disease; BMI, body mass index

Variable	Categories	N (%)
Age (years)	25-35	47 (16.2)
	36-45	68 (23.4)
	46-55	92 (31.6)
	≥56	84 (28.9)
Gender	Female	127 (43.6)
	Male	164 (56.4)
Nationality	Saudi	265 (91.1)
	Non-Saudi	26 (8.9)
Place of residence	Rabigh	216 (74.2)
	Outside Rabigh	75 (25.8)
Level of education	Illiterate	29 (10)
	Elementary and preparatory school	56 (19.2)
	High school	94 (32.3)
	Bachelors’ degree	81 (27.8)
	Postgraduate	31 (10.7)
Marital status	Married	218 (74.9)
	Non-married	73 (25)
Social class by monthly income	Low (< 5000 SR)	77 (26.5)
	Moderate (5000-10000 SR)	123 (42.3)
	High (>10000 SR)	91 (31.3)
Occupation	Employed	164 (56.4)
	Unemployed	85 (29.2)
	Retired	42 (14.4)
Smoking	Current smoker	29 (10.0)
	Ex-smoker (>1 month)	45 (15.5)
	Nonsmoking	189 (64.9)
	Passive smoker	28 (9.6)
Physical activity	Yes	29 (10)
	Regular	147 (50.5)
	Irregular	115 (39.5)
	No	0.0 (0.0)
History of chronic disease	Yes	177 (60.8)
	No	114 (39.2)
If yes, type of chronic disease	HTN	130 (44.7)
	Dyslipidemia	81 (27.8)
	CVD	39 (13.4)
	Other	83 (28.5)
BMI categories	Underweight	1 (0.3)
	Normal weight	107 (36.8)
	Overweight	125 (43)
	Obese	58 (19.9)
Weight (kg) mean ± SD		72.73 ± 13.34
Height (m) mean ± SD		1.72 ± 0.36
BMI (kg/m^2^) mean ± SD		26.88 ± 4.57
Duration of diabetes	<2 years	58 (19.9)
	2-5 years	110 (37.8)
	>5 years	123 (42.3)
Treatment	Insulin	20 (6.9)
	Oral antihyperglycemic agent	244 (83.8)
	Both	27 (9.3)
History of vitamin B12 intake	Yes	197 (67.7)
	No	94 (32.3)
If yes, what type	Injection	6 (2.1)
	Oral	191 (65.6)
History of hospitalization for high blood glucose	Yes	131 (45)
	No	160 (55)
How often do you check blood sugar?	Daily	171 (58.8)
	Once or twice / week	93 (32)
	Once or twice / month	22 (7.6)
	Never been asked to check blood glucose	5 (1.7)
Diabetes complications	CVD	41 (14.1)
	Nerve damage	144 (49.5)
	Kidney damage	37 (12.7)
	Foot damage	48 (16.5)
When was your last eye exam?	<1 year ago	164 (56.4)
	1-2 years ago	79 (27.1)
	3 to 5 years ago	26 (8.9)
	More than 5 years ago	5 (1.7)
	Never checked my eye	17 (5.8)
Have diabetic retinopathy	Yes	121 (41.6)
	No	170 (58.4)
If yes, duration of diabetic retinopathy	Less than 1 year	35 (12)
	1-2 years	42 (14.4)
	3-5 years	24 (8.2)
	More than 5 years	20 (6.9)
Have your eyes been checked by a doctor last year?	Yes	168 (57.7)
	No	123 (42.3)

Table 1 also presents the distribution of the participants according to conditions related to T2D, DR, and last eye exam. Approximately 42.3% of the participants had T2D for more than five years, 83.8% were on oral antihyperglycemic agents for T2D control, 61.2% had a history of vitamin B12 intake, and 59.5% took oral vitamin B12. Under half the population (45%) had a history of hospitalization for high blood glucose, and 58.8% reported checking their blood glucose daily. The most common T2D complications reported by the participants were nerve damage (49.5%) and foot damage (16.5%). Over half of all participants (56.4%) had their last eye exam within the past year, 41.6% had DR, and 57.7% had their eyes checked by a doctor in the last year.

Regarding the participants’ responses to DR awareness items, 81.8% heard about general complications of T2D, 73.2% heard about eye complications of T2D, and 35.1% knew the relationship between retinopathy and T2D. Approximately 45.4% of the participants knew what DR is, 39.5% knew factors leading to DR, 68.4% knew that vision could be affected due to high blood glucose levels, and 55.3% thought that T2D might lead to blindness. About one-third (30.6%) of them knew that a person with diabetes should undergo an eye checkup yearly or every two years, and 41.2% reported that when they are first diagnosed with T2D, they must screen their eyes at the time of diagnosis. Most participants (60.8%) thought DR is a treatable condition, 56.7% thought they needed regular eye screening for DR if their eyes were healthy, and 71.1% thought that good control of T2D might prevent DR (Table 2). The mean level of awareness score was 7.23 ± 2.74, and 28.9% of the population had poor awareness, 29.5% had moderate awareness, and 31.6% had good awareness of DR.

**Table 2:** Responses of the participants to knowledge items related to diabetic retinopathy (N=291).

Questions and responses	N (%)
Have you heard about general complications of diabetes?	
Yes	238 (81.8)
No	53 (18.2)
Have you ever heard about eye complications of diabetes?	
Yes	213 (73.2)
No	75 (25.8)
I don’t care	3 (1.0)
Do you know relationship between retinopathy and diabetes?	
Yes	102 (35.1)
No	177 (60.8)
I don’t care	12 (4.1)
Do you know what diabetic retinopathy is?	
Yes	132 (45.4)
No	152 (52.2)
I don’t care	7 (2.4)
Do you know the factors that lead to diabetic retinopathy?	
Yes	115 (39.5)
No	169 (58.1)
I don’t care	7 (2.4)
Do you know if vision can be affected by high blood sugar levels?	
Yes	199 (68.4)
No	87 (29.9)
I don’t care	5 (1.7)
Do you think diabetes may lead to blindness?	
Yes	161 (55.3)
No	126 (43.3)
I don’t care	4 (1.4)
How frequently should a person with diabetes undergo an eye checkup?	
I don't know	29 (10)
Every 6 months	122 (41.9)
Yearly or every 2 years	89 (30.6)
Only when vision is affected	51 (17.5)
When you are first diagnosed with diabetes, you must screen your eyes	
At time of diagnosis	120 (41.2)
5 years after the diagnosis	41 (14.1)
If there are eye symptoms only	58 (19.9)
I don't know	72 (24.7)
Do you think retinopathy is a treatable condition?	
Yes	177 (60.8)
No	43 (14.8)
I don’t care	71 (24.4)
I don't think that I need a regular eye screening for diabetic retinopathy if my eyes are healthy	
Yes	165 (56.7)
No	126 (43.3)
Do you think good control of diabetes might prevent diabetic retinopathy?	
Yes	207 (71.1)
No	72 (24.7)
I don’t care	12 (4.1)
level of awareness score	
Poor	28.9%
Moderate	39.5%
Good	31.6%

Table 3 shows that participants who are aged 46-55 years, female, reside in Rabigh, have a master’s degree, earn >10000 SR monthly, regularly practice physical activity, and have no history of chronic diseases had a significantly higher DR level of awareness score than others (p<0.05). A nonsignificant relationship was found between participants’ nationality, marital status, occupation, smoking status, and BMI categories and the level of knowledge about DR (p>0.05).

**Table 3:** Relationship between knowledge level of diabetic retinopathy and participants’ sociodemographic characters, special habits, chronic diseases, and conditions related to type 2 diabetes. SR, Saudi Riyals; BMI, body mass index *Significant at p≤0.05

Variable	Categories	Awareness level			P-value Pearson Chi-square
		Poor, N (%)	Fair, N (%)	Good, N (%)	
					<0.0001*
Age (years)	25-35	13 (27.7)	15 (31.9)	19 (40.4)	
	36-45	18 (26.5)	24 (35.3)	26 (38.2)	
	46-55	17 (18.5)	36 (39.1)	39 (42.4)	
	≥ 56	36 (42.9)	40 (47.6)	8 (9.35)	
Gender	Female	26 (20.5)	53 (41.7)	48 (37.8)	0.01*
	Male	58 (35.4)	62 (37.8)	44 (26.8)	
Nationality	Saudi	73 (27.5)	106 (40)	86 (32.5)	0.27
	Non-Saudi	11 (42.3)	9 (34.6)	6 (23.1)	
Place of Residence	Rabigh	65 (30.1)	76 (35.2)	75 (34.7)	0.03*
	Outside Rabigh	19 (25.3)	39 (52)	17 (22.7)	
Level of education	None	13 (44.8)	13 (44.8)	3 (10.3)	<0.0001*
	Elementary and preparatory	25 (44.6)	23 (39.3)	9 (16.1)	
	High school	23 (24.5)	36 (38.3)	35 (37.2)	
	Bachelor’ degree	19 (23.5)	32 (39.5)	30 (37)	
	Diploma	4 (25)	6 (37.5)	6 (37.5)	
	Masters’ degree	0 (0.0)	6 (40)	9 (60)	
Marital status	Single	8 (33.3)	10 (41.7)	6 (25)	0.40
	Married	60 (27.5)	87 (39.9)	71 (32.6)	
	Widowed	15 (40.5)	11 (29.7)	11 (29.7)	
	Divorced	1 (8.3)	7 (58.3)	4 (33.3)	
Monthly income (SR)	< 5000	25 (32.5)	37 (48.1)	15 (19.5)	0.01*
	5000-10000	38 (30.9)	50 (40.7)	35 (28.5)	
	>10000	21 (23.1)	28 (30.8)	42 (46.2)	
Occupation	Employed	46 (28)	61 (37.2)	57 (34.8)	0.09
	Unemployed	21 (24.7)	35 (41.2)	29 (34.1)	
	Retired	17 (40.5)	19 (45.2)	6 (14.3)	
Smoking	Current smoker	10 (34.5)	14 (48.3)	5 (17.2)	0.16
	Ex-smoker (>1 month)	12 (26.7)	22 (48.9)	11 (24.4)	
	Nonsmoking	58 (30.7)	66 (34.9)	65 (34.4)	
	Passive smoker	4 (14.3)	13 (46.4)	11 (39.3)	
Physical activity	Regular	9 (31)	6 (20.7)	14 (48.3)	0.05*
	Irregular	39 (26.5)	57 (38.8)	51 (34.7)	
	No	36 (31.3)	52 (45.2)	27 (23.5)	
History of chronic disease	Yes	56 (31.6)	78 (44.1)	43 (24.3)	<0.0001*
	No	28 (24.6)	37 (32.5)	49 (43)	
BMI Categories	Underweight	0 (0.0)	48 (44.9)	1 (100)	0.45
	Normal weight	31 (29)	42 (33.6)	28 (26.2)	
	Overweight	38 (30.4)	24 (41.4)	45 (36)	
	Obese	15 (25.9)	0 (0.0)	19 (32.8)	
Duration of diabetes	<2 years	15 (25.9)	22 (37.9)	21 (36.2)	0.07
	2-5 years	26 (23.6)	41 (37.3)	43 (39.1)	
	>5 years	43 (35)	52 (42.3)	28 (22.8)	
Treatment of diabetes	Insulin	6 (30)	7 (35)	7 (35)	0.92
	Oral antihyperglycemic agent	72 (29.5)	97 (39.8)	75 (30.7)	
	Both	6 (22.2)	11 (40.7)	10 (37)	
History of vitamin B12 intake	Yes	52 (29.2)	66 (37.1)	60 (33.7)	0.51
	No	32 (28.3)	49 (43.4)	32 (28.3)	
History of hospitalization for high blood glucose	Yes	44 (33.6)	52 (39.7)	35 (26.7)	0.16
	No	40 (24.5)	63 (39.4)	57 (35.5)	
How often do you check blood glucose?	Daily	43 (25.1)	70 (40.9)	58 (33.9)	0.22
	Once or twice / week	34 (36.6)	34 (36.6)	24 (26.9)	
	Once or twice / month	7 (31.8)	7 (31.8)	8 (36.4)	
	Never been asked to check blood glucose	0 (0.0)	4 (80)	1 (20)	
Have diabetic retinopathy	Yes	43 (35.2)	52 (42.6)	27 (22.1)	0.01*
	No	41 (23.8)	66 (38.4)	65 (37.8)	
Duration of diabetic retinopathy (years)	<1	7 (20)	18 (51.4)	10 (28.6)	0.01*
	1-2	16 (38.1)	14 (33.3)	12 (28.6)	
	3-5	12 (48)	9 (36)	4 (16)	
	>5	8 (40)	11 (55)	1 (5)	
Were your eyes been checked by a doctor last year?	Yes	34 (20.2)	66 (39.3)	68 (40.5)	<0.0001*
	No	50 (40.7)	49 (39.8)	24 (19.5)	

Table 3 also showed that participants without DR or who had DR for less than two years and those who had their eyes checked by a doctor last year had a significantly higher DR level of awareness score (p<0.05). We found a nonsignificant relationship between the level of participants’ DR level of awareness and duration of T2D, type of T2D treatment, history of vitamin B12 intake, history of hospitalization for high blood glucose, and frequency of checking blood glucose (p>0.05).

**Figure 1:**
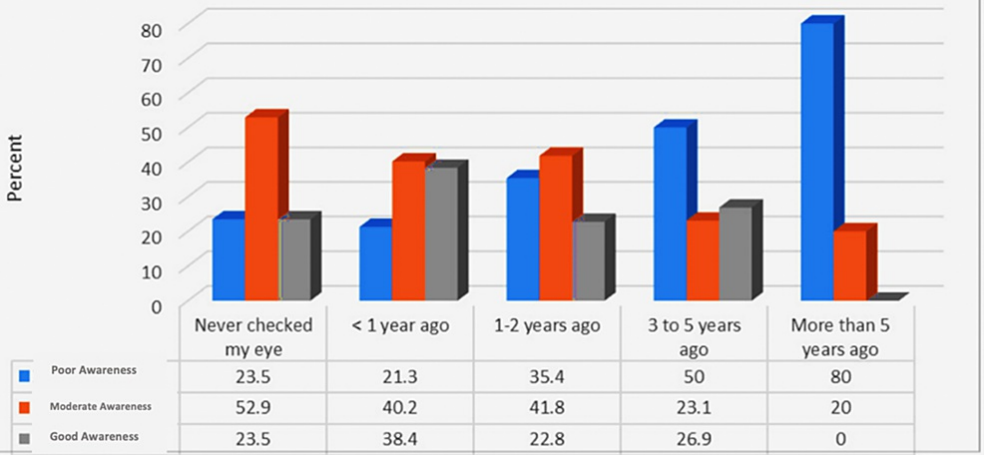
Relationship between awareness level of DR and the time of the last eye exam. Note: (χ2=22.73; p=0.004) DR, diabetic retinopathy

Participants who had their last eye examination less than one year ago had a significantly higher level of awareness regarding DR (Figure 1; p<0.05).

## Discussion

Our study explored patient awareness of DR and related risk factors in patients visiting an outpatient clinic in the general hospital in Rabigh. We found that 39.5% of patients with T2D had moderate levels of awareness regarding DR, 31.6% had good levels of awareness, and 28.9% had poor levels of awareness. Several studies have documented the levels of awareness of T2D patients regarding DR. In a study in Tabuk, 47.1% had poor levels of awareness, 27.7% had moderate levels of awareness, and 25.1% had good levels of awareness [18]. In another study in Jeddah, 82.6% were aware of DR [19]. This difference could be related to a range of socioeconomic factors and geographic location. Most of the patients in our study (n=213; 73.2%) were aware that T2D could impair the eyes, and 55.3% thought DR could lead to blindness. In Tabuk, 86.9% of patients were aware that diabetes could affect the eyes, and 78.5% reported that DR could lead to blindness [18]. These results were similar to a study conducted in Jordan in which 88.2% of patients with diabetes were aware that diabetes could affect the eyes, and 81% thought DR could lead to blindness [20].

The differences in awareness are often due to different educational levels. Most patients in our study believed that good control of their diabetes would prevent DR, but our population had relatively lower awareness than other studies done in Tabuk (94%) and Jordan (82.7%); this indicates a lack of awareness and the necessity for programs to improve awareness in our population [18, 20]. Patients were asked how often they thought their eyes should be checked after being diagnosed with diabetes, and 41.9% said every six months, while 30.6% answered yearly or every two years, and 17.5% said only when vision is affected. Nearly half of respondents in Jordan (50.6%) answered every six months, 20.7% answered every year, 5.1% answered every two years, and 23.6% answered that retinal assessment is important only when vision is affected [20]. Some 60.8% were aware of the existence of the treatment of DR. These findings were lower in comparison to the studies done in Malaysia (72.3%) and Tabuk (86.1%) [18, 21]. These variances could be due to differences in sample size and sampling procedure.

Our study had some limitations, being a single-center study with small number of patients. This impacts the generalizability of our results to other populations. We assess the level of awareness only in one type of diabetes. Moreover, there was no tools or education session to improve the level of awareness.

## Conclusions

In this study, a sample of patients with T2D were studied to assess their level of DR awareness. Most patients reported moderate levels of awareness. This result emphasizes the need for increased awareness about T2D, DR, and its prevention. Strategies to develop and implement awareness initiatives are crucial, and patients need to be motivated for retinal screening to reduce the risk of visual complications. To reach undiagnosed people, DR screening programs should not be limited to eyecare centers; screening campaigns should be implemented near their homes. Future national multi-center studies having larger number of patients are recommended. 
